# KLHL1 Controls Ca_V_3.2 Expression in DRG Neurons and Mechanical Sensitivity to Pain

**DOI:** 10.3389/fnmol.2019.00315

**Published:** 2020-01-08

**Authors:** Elizabeth Martínez-Hernández, Alissa Zeglin, Erik Almazan, Paula Perissinotti, Yungui He, Michael Koob, Jody L. Martin, Erika S. Piedras-Rentería

**Affiliations:** ^1^Department of Cell and Molecular Physiology, Loyola University Chicago, Maywood, IL, United States; ^2^Stritch School of Medicine, Loyola University Chicago, Maywood, IL, United States; ^3^Neuroscience Division of the Cardiovascular Institute, Loyola University Chicago, Maywood, IL, United States; ^4^Institute for Translational Neuroscience, University of Minnesota, Minneapolis, MN, United States; ^5^Department of Laboratory Medicine & Pathology, University of Minnesota, Minneapolis, MN, United States

**Keywords:** voltage-gated calcium channel, T-type channel, mechanical sensitivity, pain control, KLHL1, CaV3.2, DRG, shRNA

## Abstract

Dorsal root ganglion (DRG) neurons process pain signaling through specialized nociceptors located in their peripheral endings. It has long been established low voltage-activated (LVA) Ca_V_3.2 calcium channels control neuronal excitability during sensory perception in these neurons. Silencing Ca_V_3.2 activity with antisense RNA or genetic ablation results in anti-nociceptive, anti-hyperalgesic and anti-allodynic effects. Ca_V_3.2 channels are regulated by many proteins (Weiss and Zamponi, 2017), including KLHL1, a neuronal actin-binding protein that stabilizes channel activity by recycling it back to the plasma membrane through the recycling endosome. We explored whether manipulation of KLHL1 levels and thereby function as a Ca_V_3.2 modifier can modulate DRG excitability and mechanical pain transmission or sensitivity to pain. We first assessed the mechanical sensitivity threshold and DRG properties in the KLHL1 KO mouse model. KO DRG neurons exhibited smaller T-type current density compared to WT without significant changes in voltage dependence, as expected in the absence of its modulator. Western blot analysis confirmed Ca_V_3.2 but not Ca_V_3.1, Ca_V_3.3, Ca_V_2.1, or Ca_V_2.2 protein levels were significantly decreased; and reduced neuron excitability and decreased pain sensitivity were also found in the KLHL1 KO model. Analogously, transient down-regulation of KLHL1 levels in WT mice with viral delivery of anti-KLHL1 shRNA also resulted in decreased pain sensitivity. These two experimental approaches confirm KLHL1 as a physiological modulator of excitability and pain sensitivity, providing a novel target to control peripheral pain.

## Introduction

Nociceptive pathways are generally activated in response to noxious stimuli as protection from injury, yet chronic pain induces allodynia and hyperalgesia due to primary dysfunction, usually caused by nerve injury. Long-term changes triggered by nerve injury include altered gene expression in Dorsal root ganglion (DRG) and the spinal cord (Choi et al., 2007; Basbaum et al., 2009; Bourinet et al., 2016). The role of low voltage-activated (LVA) calcium Ca_V_3.2 channels in pain sensation is well established; they contribute to nociception by lowering the threshold for action potential (AP) in DRG neurons (White et al., 1989; Cain and Snutch, 2010; Todorovic and Jevtovic-Todorovic, 2011). LVA channels (also called T-type) are comprised of Ca_V_3.1, Ca_V_3.2, and Ca_V_3.3 channels; their biophysical properties such as the relatively small depolarization required for their activation, and window currents confer them the capability to act as burst firing modulators (Cribbs et al., 1998; Perez-Reyes et al., 1998; Bourinet et al., 2016).

T-type currents are up-regulated in various models of chronic pain, such as chronic constriction injury, spinal nerve ligation, STZ-diabetes, the carregin pain model, and drug-induced diabetic neuropathy (Jagodic et al., 2007, 2008; Melrose et al., 2007; Takahashi et al., 2010; Marger et al., 2011; Watanabe et al., 2015; Li et al., 2017; Bellampalli et al., 2019). Ca_V_3.2 can be upregulated by increased expression of USP5, which interacts with and de-ubiquitinates these channels thereby decreasing their degradation (Garcia-Caballero et al., 2014; Stemkowski et al., 2016). Moreover, treatment with T-type channel blockers results in reduced mechanical hyperalgesia in the spinal nerve ligation model (Dogrul et al., 2003; Chen et al., 2015). Similarly, manipulation of the expression levels of Ca_V_3.2, as in the KO mouse model (Choi et al., 2007) or by selective knockdown in DRG neurons using antisense (Bourinet et al., 2005) results in attenuated pain responses, confirming the critical role of Ca_V_3.2 in pain transmission.

Ca_V_3.2 are therefore viable pharmacological targets to control pain (Dogrul et al., 2003; Flatters and Bennett, 2004; Okubo et al., 2011; Chen et al., 2015). An alternative strategy to modulate channel function is to target auxiliary or modulatory subunits to indirectly affect channel activity or trafficking to the plasma membrane (Weiss and Zamponi, 2019). This approach has been highly successful in the modulation of high-voltage-activated (HVA) channels by targeting of the α_2_δ subunit. This auxiliary subunit enhances Ca^2+^ currents in part by modulating Ca_V_ trafficking, altering their density and kinetics (Davies et al., 2007; Hendrich et al., 2008) and is one of the molecular targets of the antiepileptic and analgesic drug Gabapentin (GBP; Gee et al., 1996; Suárez et al., 2005; Martins et al., 2015). Consequently, treatment with GBP results in a significant decrease of N-type Ca_V_2.2 currents due to a reduction of functional channels at the plasma membrane (Vega-Hernández and Felix, 2002; Hendrich et al., 2008).

Here, we targeted a protein that affects Ca_V_3.2 trafficking to modulate its function and excitability in DRG neurons. Our target is Kelch-like 1 (KLHL1), a structural protein that binds to Ca_V_3.2 and actin and alters Ca_V_3.2 function. KLHL1’s primary effect on Ca_V_3.2 recycle it back to the plasma membrane *via* direct association with the channel and actin filaments, thus preventing its degradation; this process is mediated through increased recycling endosome-mediated channel insertion in the plasma membrane and results in an increased number of functional channels and ultimately increased Ca_V_3.2-mediated T-type current density. KLHL1 also remains bound to Cav3.2 and F-actin at the plasma membrane, altering the channel kinetics of deactivation (Aromolaran et al., 2009, 2010, 2012). Here, we show that the expression levels of the structural protein KLHL1 can be altered to manipulate DRG neuron excitability and mechanical sensitivity in mice.

## Materials and Methods

### Cell Culture

DRG cultures were obtained as described (Gandini et al., 2014). In brief, DRG were dissected from C57BL/6 mice (P6-P10) in Advanced DMEM Medium (Gibco) supplemented with 20% of Fetal Bovine Serum (Gibco), washed, and digested for 40 min at 37°C with a mixture of trypsin type XI (1.25 mg/ml, Sigma) and collagenase IV (1.25 mg/ml, Sigma), followed by mechanical dissociation. Cells were spun down at 1,000 g for 5 min at 10°C and re-suspended in Advanced DMEM medium supplemented with 10% FBS. Cells were plated onto L-lysine-covered coverslips (12 mm, Carolina Biological Supply, Burlington, NC, USA) and kept in a 5% CO_2_ humidified atmosphere at 37°C. The Patch-clamp recordings were made 24 h after dissociation (1 day *in vitro*, 1 DIV).

### Biochemistry

Western blots. Crude protein was extracted from at least three WT or KLHL1 KO DRG ganglia pooled together and separated by SDS-PAGE electrophoresis (8%, at 100 V for 90 min) for transfer to a PVDF membrane (BioRad). Membranes were washed in Tris-buffered saline (TBS) supplemented with 0.05% Tween 20 (TBST) and blocked for 1 h in TBST-5% milk at room temperature (Florio et al., 1992). Membranes were incubated at 4°C overnight with primary antibodies against Ca_V_3.1 (1:1,000, Millipore, CA, USA), Ca_V_3.2 (1:2,000, Santa Cruz Biotechnology, Inc., Santa Cruz, CA, USA), Ca_V_3.3 (1:1,000, Alomone), Ca_V_2.1 (1:1,000, Alomone) or Ca_V_2.2 (1:1,000, Alomone). GAPDH (Santa Cruz Biotechnology, Inc., Santa Cruz, CA, USA; 1:1,000) was used as an internal reference to normalize for protein loading. Horseradish peroxidase (HRP)-conjugated secondary antibodies were used for detection (1:2,000; Pierce) with Supersignal Femto (Pierce, IL, USA) using a ChemiDoc MP System (BioRad).

Immunoprecipitation. Crude membrane preparations were obtained using standard protocols (Aromolaran et al., 2010); a fraction of the sample was reserved prior to immunoprecipitation (input, 30× less concentrated than the IP samples) and the remaining volume was divided up for all experiments. Samples were processed by addition of primary antibodies (Ca_V_3.2, 1:40 and KLHL1, 1:40, Santa Cruz Biotechnology, or IgG, 1:40, Alpha Diagnostic Intl. Inc., San Antonio, TX, USA) and incubated for 1–3 h at 4°C followed by overnight incubation with protein A/G agarose beads (Biovision, Mountain View, CA, USA) on a shaking plate at 4°C. Samples were washed and processed for western blot analysis as described.

### Electrophysiology

Whole-cell patch-clamp recordings were obtained at 1DIV using an Axopatch 200B amplifier (Axon Instruments, Union City, CA, USA) at room temperature. Data were acquired at 1 kHz and digitized at 20 kHz. Calcium currents were recorded using an external solution containing (in mM) 5 CaCl_2_, 140 TEA-Cl, 10 HEPES and 10 glucose (pH 7.4, 300 mosmol/kgH_2_O). The intracellular solution contained (in mM): 108 CsMeSO_3_, 4 MgCl_2_, 10 Cs-EGTA, 9 HEPES, 5 ATP-Mg, 1 GTP-Li and 15 phosphocreatine-Tris. Pipette resistances were 3.0–4.0 MΩ. Series resistance (Rs) was compensated online (>80%), only cells with *Rs* <15 MΩ were used. Data were acquired and analyzed using pClamp10 software (Molecular Devices).

Total currents were elicited using depolarizing steps (test potentials, *V*_T_) from −60 to +60 mV (Δ*V* = 10 mV) from a holding potential (*V*_H_) of −90 mV. HVA currents were obtained from *V*_H_ = −50 mV to *V*_T_ = −60 to +60 mV (Δ*V* = 10 mV). HVA currents traces were subtracted from the total current traces at each *V*_H_ to obtain the LVA current component.

APs were measured using an external solution containing (in mM): 135 NaCl, 5 KCl, 2 CaCl_2_, 1 MgCl_2_, 10 HEPES, 10 glucose; the intracellular solution composition was (in mM) 110 K-gluconate, 20 KCl, 2 MgCl_2_, 1 EGTA, 10 HEPES, 2 ATP-Mg, 0.25 GTP-Li and 10 phosphocreatine-Tris. The APs were triggered by four consecutive 1.5-s-long current depolarizing ramps at 20, 40, 60, or 80 pA/s. Rheobase was determined as the minimum current necessary to elicit an AP from a membrane potential of −75 mV.

### Viral Production

Adeno-associated viral constructs containing small hairpin RNAs (shRNA) targeting KLHL1 were designed; shKLHL1-AAV contained two sequences from the mouse gene (NM_053105.2) spanning nucleotides (nt) 1,812–1,830 and 2,121–2,139 (GGCCAGTGATGATGTAAAT and GGGAATGGATAATAACAAA, respectively); these segments were synthesized and cloned into an AAV shuttle vector pZacf-U6-Luc-Zsgreen (U Penn Gene Therapy Core) as described before (Zolotukhin et al., 1999; Sarkey et al., 2011). These shuttle plasmids, along with pHelper and pAAV2/8 were transfected into AAV-293 cells using the Virapack transfection kit (Stratagene) and purified by an iodixanol step gradient as described before (Pradhan et al., 2010). Titer was assessed by serial dilutions of virus and infection of HT1080 cells. AAV particle titer was quantified by SDS-PAGE (Zolotukhin et al., 1999; Kohlbrenner et al., 2012). EGFP-AAV was generated in house by the same method.

### AAV Injections

All animal protocols used in this study were reviewed and approved by an independent Institutional Animal Care and Use Committee (IACUC). Hind paws of 13- weeks old WT male mice were injected with a control virus (EGFP-AAV, shCtrl,) or shKLHL1-AAV under blind conditions. The summary of the experimental conditions used is depicted in Figure 6A. The initial trial (*n* = 7) received 4.2 × 10^10^ shKLHL1-AAV or 5.5 × 10^10^ EGP-AAV vector genomes. The second trial (*n* = 11) received a high titer, 9.0 × 10^10^ shKLHL1-AAV or EGFP-AAV vector genomes over 2 days. Viruses were diluted such that each individual injection volume was 5 μl total. Mice were given pain medication (Buprenorphine, 0.05 mg/kg, s.c.) for the first 2 days following the last injection and were allowed to recover in observation for 4–5 days while checked for any limp or lameness; all mice were confirmed healthy after injections.

**Figure 1:**
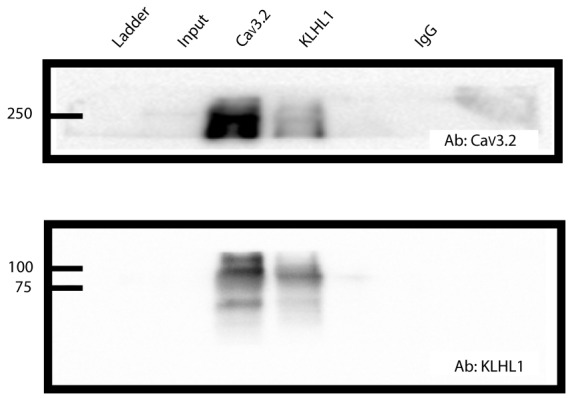
Ca_V_3.2 and KLHL1 interact in mice dorsal root ganglion (DRG) ganglia. Co-immunoprecipitation of Ca_V_3.2 and KLHL1 using antibodies against Ca_V_3.2 (top) and KLHL1 (bottom).

**Figure 2:**
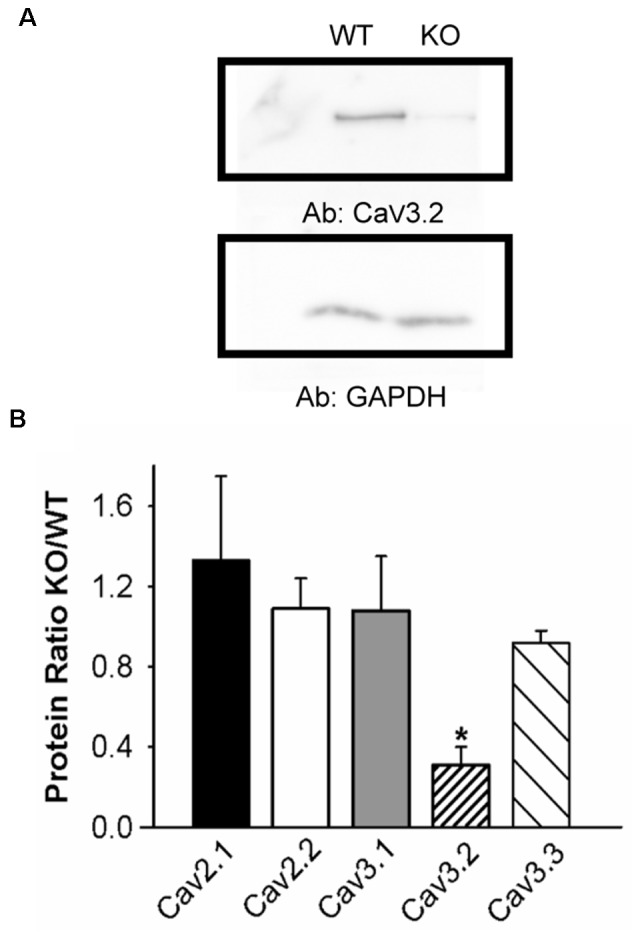
Lower Ca_V_3.2 expression in KLHL1 KO DRG. **(A)** Western Blot example of Ca_V_3.2 expression. GAPDH was used as loading control. **(B)** Densitometric quantification of voltage-gated calcium channel protein levels by Western Blot analysis of high-voltage-activated (HVA; Ca_V_2.1 and Ca_V_2.2) and low voltage-activated (LVA; T-type; Ca_V_3.1, Ca_V_3.2, and Ca_V_3.3) α subunits. Protein levels are expressed as KO/WT ratio ± SEM (*n* = 3; Ca_V_2.1 and Ca_V_3.2, *n* = 4; *p < 0.05, student’s *t*-test).

**Figure 3:**
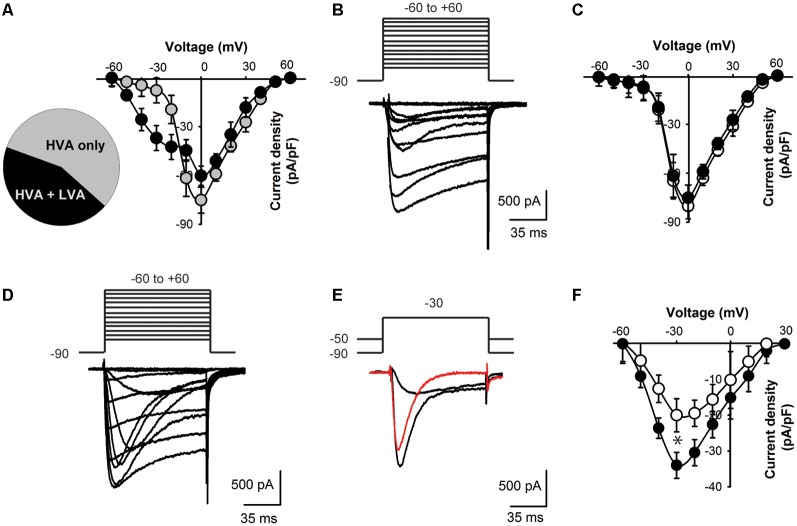
Lower T-type current density in KLHL1 KO DRG neurons. **(A)** Distribution of current profiles found in WT DRG neurons and their I–V relationships: 56% neurons expressed only HVA currents (black symbols) and 44% expressed a mixture of LVA + HVA currents (gray symbols). **(B)** Representative Ca^2+^ currents recorded from a small DRG neuron (23 pF) expressing HVA currents only; the voltage protocol is shown above the traces (mV). **(C)** I–V curves for HVA-only currents recorded from WT (black) and KO neurons (white), *V*_H_ = −90 mV. **(D)** Representative traces of a neuron displaying both LVA and HVA Ca^2+^ currents. **(E)** Representative LVA current trace (red) obtained by current subtraction. **(F)** I-V curves of the subtracted LVA component for WT (black) and KO (white; *n* = 10, 11; *p < 0.004).

**Figure 4:**
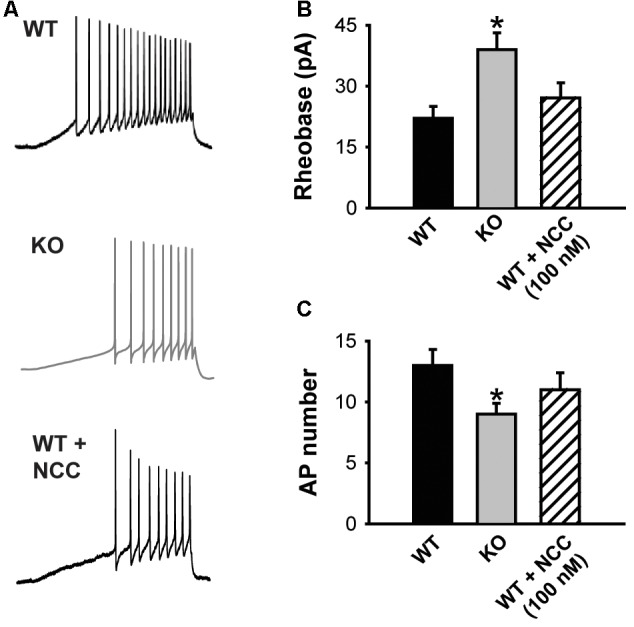
The absence of KLHL1 alters DRG neuron excitability. **(A)** Examples of action potential (AP) trains generated by a depolarizing ramp rate of 60 pA/s in WT, KLHL1-KO and WT + 100 nM of NCC 55–0396 DRG neurons. **(B)** Rheobase values. **(C)** Average number of APs (WT, *n* = 10, KO, *n* = 11, WT + NCC, *n* = 7; *p = 0.003).

**Figure 5:**
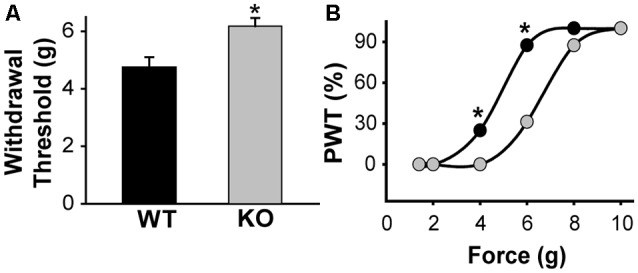
KLHL1 KO mice are less sensitive to mechanical stimulation. **(A)** Paw withdrawal threshold in response to mechanical stimulation, expressed as g of force (*n* = 20, WT and KO; *p < 0.05). **(B)** Data expressed as cumulative % of the population of mice responding at a given force; PWT, paw withdrawal threshold (*p < 0.05, non-parametrical Kolmogorov–Smirnov test).

**Figure 6:**
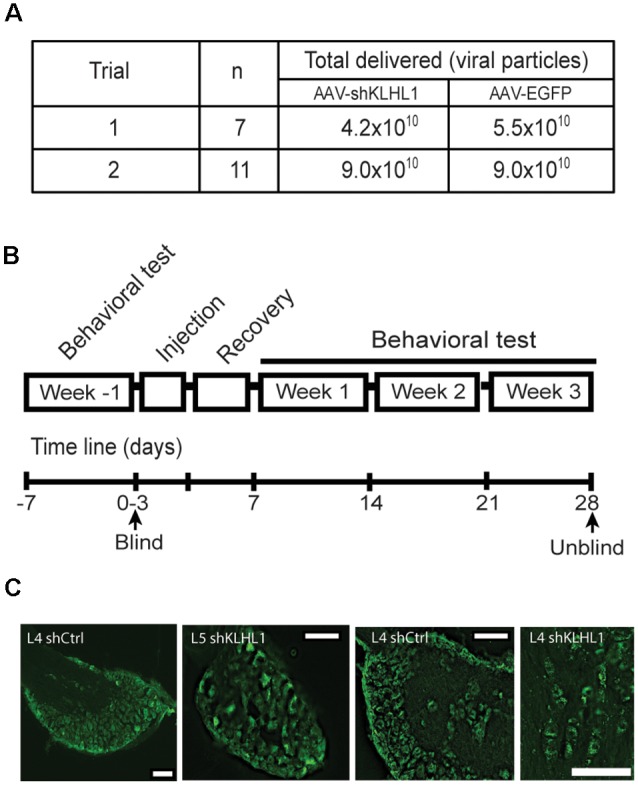
KLHL1 knockdown with shKLHL1-AAV leads to Ca_V_3.2 down-regulation. **(A)** Experimental conditions used. **(B)** Timeline of behavioral experiments. **(C)** Example of DRG slices from mouse injected with control EGFP-AAV and shKLHL1-EGFP AAV; size bar, 100 μM.

Behavioral tests were performed twice a week (and averaged) for a total of 3 weeks after injections. Baseline withdrawal threshold responses were determined for 1 week before injections.

% Paw-withdrawal threshold was reported as the % of mice in the total population displaying withdrawal thresholds at all forces tested.

### Von Frey Filament Tests

Hind paw withdrawal experiments were carried out in male mice ~16 weeks old; animals had access to food and water *ad libitum*, all experiments followed IACUC-approved standard procedures. Only males were used in the first study because our preliminary data show male KLHL1 KO mice display a clear phenotype in contrast with females, where differences are more difficult to establish if present. Mice were placed on a wire mesh-bottom testing apparatus and allowed to acclimate for 15 min before assessing mechanical allodynia. Measurements were recorded by applying von Frey filaments (North Coast, Morgan Hill, CA, USA) ranging from 1.4 to 10 g to the plantar surface of the mouse hind paw; each filament was assessed for a total of five consecutive times. Hind paw withdrawal response times of less than 2 s were considered positive. The withdrawal threshold was calculated as the filament force at which each mouse had a positive response more than three times out of five (Chaplan et al., 1994; Bonin et al., 2014).

### DRG Slices

Mice were anesthetized with isoflurane before euthanasia; DRG were excised and fixed with 4% paraformaldehyde for 4 h, cryoprotected overnight (30% sucrose in PBS), embedded in OCT (Tissue Tek, Fisher Scientific, Hampton, NH, USA), and frozen with dry ice. 30 μm-thick sections were cut using a cryostat and mounted onto Superfrost Plus slides (Fisher Scientific, Hampton, NH, USA). Slides were washed three times with PBS-glycine, dried and protected with coverslips. Fluorescence images were captured using IX80 Olympus inverted epifluorescence microscope using a 10× objective and analyzed using deconvolution.

### Statistical Analysis

Statistical analysis was performed with SigmaPlot 11 Software. Statistical significance was determined as *P* < 0.05, using student’s *t*-test or Kolmogorov–Smirnov non-parametric analysis (Kolmogorov–Smirnov test). Results are presented as mean ± SEM.

## Results

### KLHL1 Is Expressed and Interacts With Ca_V_3.2 in DRG Neurons

Co-immunoprecipitation of Ca_V_3.2 and KLHL1 is detected in overexpression experiments in HEK-293 cells and in whole brain samples, demonstrating direct interaction between these two proteins (Aromolaran et al., 2009, 2010, 2012). Figure 1 shows an example of pull-down experiments from DRG protein extracts using Ca_V_3.2 (top) or KLHL1 antibodies (bottom, IgG was used as negative control). These data confirm the presence of KLHL1 in DRG neurons and its interaction with Ca_V_3.2 T-type channels.

### KLHL1 KO DRG Neurons Exhibit Low Ca_V_3.2 Channel Expression and Reduced Excitability

We next assessed the effect of KLHL1 deletion on Ca_V_ expression in DRG neurons. HVA channel expression was statistically similar in both KLHL1 KO and WT mice, as seen in Figure 2, which shows the KO/WT protein ratio for Ca_V_2.1 (1.3 ± 0.4, *n* = 4) and Ca_V_2.2 (1.0 ± 0.1, *n* = 3). In contrast, Ca_V_3.2 expression was statistically lower among LVA channels (0.3 ± 0.09, *n* = 4) in the KLHL1 KO tissue (*p* = 0.04) whereas Ca_V_3.1 and Ca_V_3.3 expression remained constant (1.0 ± 0.2, *n* = 3; and 0.9 ± 0.06, *n* = 4 respectively). Thus, the absence of KLHL1 results in decreased Ca_V_3.2 protein expression, which remains uncompensated for in the adult KLHL1 KO mice DRG.

To assess the physiological impact of lower Ca_V_3.2 expression in the absence of KLHL1 we analyzed Ca^2+^ current densities in DRG neurons. We found two neuronal populations according to the Ca^2+^ currents they expressed (Figure 3A): 56% of all WT neurons elicited only HVA currents (capacitance = 23.0 ± 3.1 pF, *n* = 14; gray symbols); the remaining cells (44%) expressed both HVA and LVA currents. The latter group had an average capacitance of 18 pF ± 2.4 pF (black symbols, *n* = 11); this value was not statistically different from HVA-only neurons (*p* = 0.1).

Overall the HVA-only population was identical between WT and KLHL1 KO neurons (56% and 53% of the total population, respectively), with current densities of 45.1 ± 5.9 and 50.1 ± 7.9 pA/pF (*n* = 10 and 13 (WT, KO); *p* = 0.1). Figure 3B depicts an example of a recording from a neuron displaying HVA currents-only. Figure 3C shows the I-V curves for WT (black circles) and KO HVA-only neurons (white circles; *n* = 10, 13; *p* = 0.2).

Neurons expressing LVA+HVA currents represented 44% of the total population in WT vs. 47% in KO neurons (*p* = 0.1). Figure 3D depicts an example of HVA+LVA currents; the rapidly inactivating LVA current component is noticeable at lower voltages. Figure 3E shows an example of current traces recorded at *V*_T_ = −30 mV from *V*_H_ = −90 and from −50 mV, respectively, which when subtracted yield the LVA current component (red trace). Figure 3F shows the LVA-only current I–V curves from WT and KO DRGs. The peak LVA current was −37.0 ± 3.6 pA/pF (*n* = 11) in WT compared to −23.0 ± 4.6 pA/pF (*n* = 10) in the KO (at −30 mV; *p* = 0.004). We studied small neurons with capacitances ranging from 18 to 28 pF to ensure only nociceptor neurons were analyzed (Andrade et al., 2010), given that D-Hair cells also display a high density of T-type currents (Dubreuil et al., 2004; Coste et al., 2007; Bernal Sierra et al., 2017), but their capacitance ranges from ~39 to 65 pA (Coste et al., 2007).

The impact of decreased Ca_V_3.2 channel expression on DRG neuron excitability was assessed using current clamp experiments. Figure 4A shows representative traces of the APs elicited by a depolarizing current ramp delivered at 60 pA/s. The KLHL1 KO neuron rheobase was significantly larger (39.0 ± 4.1 pA) than WT (22.1 ± 2.9 pA; *n* = 9, 11, *p* = 0.03; Figure 4B), in line with a reduction in LVA calcium channel expression. This increase was accompanied by a concomitant reduction in action potential number (AP; 9.2 ± 1.1, *n* = 9) compared to WT (13.1 ± 1.4, *n* = 11, *p* = 0.03; Figure 4C). This rheobase difference was abolished by application of a low dose (100 nM) of NCC 55–0396 (NCC) to partially block T-type channels in WT neurons (27.2 ± 3.7 pA, *n* = 8, *p* = 0.003).

### KLHL1 Mice Display Increased Mechanical Sensitivity Threshold

Our data shows that Ca_V_3.2 expression is down-regulated in the absence of KLHL1; KO DRG neurons display significantly lower neuronal excitability, which may, in turn, alter pain sensation. We assessed the responses to mechanical stimulation by measuring paw withdrawal thresholds in WT and KLHL1 KO mice using von Frey filaments. KLHL1 KO mice displayed significantly higher withdrawal threshold (6.1 ± 0.2 g, *n* = 20) compared to WT mice (4.7 ± 0.3 g, *n* = 20; *p* = 0.009; Figure 5A). Non-parametric analysis of paw withdrawal threshold responses within the mice population demonstrates statistical differences at 4 and 6 g of force (*p* < 0.05; Figure 5B). Thus, decreased Ca_V_3.2 expression in KLHL1 KO DRG neurons results in decreased excitability and altered pain sensitivity, confirming KLHL1 is a physiological modulator of Ca_V_3.2 in sensory neurons.

### Modulation of KLHL1 Expression Levels Alters Mechanical Sensitivity in WT Mice

Induction of excitability changes by the manipulation of KLHL1 levels could represent a novel method in the regulation of Cav3.2 expression. Therefore, we tested whether knockdown of KLHL1 expression alters mechanical sensitivity in WT mice by injecting adeno-associated viral particles (AAV) containing shRNA designed against KLHL1 into the mice hind paws (US Patent 10,047,377).

Preliminary data from neuronal cultures indicated that titers ~5.0 × 10^10^ shRNA-containing viral particles appeared to be less efficient *in vitro*. Therefore, we carried out two blind trials assessing the effect of two titer viral loads (4.2 × 10^10^ vs. 9.0 × 10^10^ viral particles; Figure 6A). All trials were performed following the timeline showed in Figure 6B and described in “Materials and Methods” section.

Figure 6C shows representative images of DRG slices obtained from WT mice injected with EGFP-AAV or shKLHL1-AAV, confirming successful delivery and uptake of the AAV. Ca_V_3.2 and KLHL1 levels from protein samples pooled from three L4 DRGs ipsilateral to the shKLHL1-AAV-injected paw were analyzed by western blot (sh). L4 DRG ipsilateral to the EGFP-AAV injection were also collected as a negative control (Ctrl; Liu et al., 2019).

**Figure 7:**
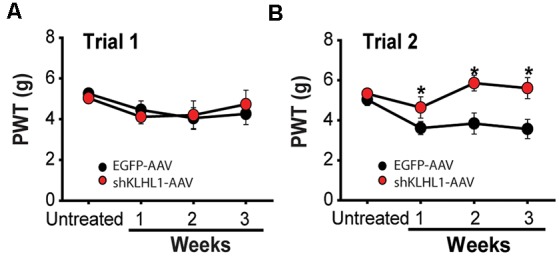
KLHL1 knockdown decreases mechanical sensitivity. **(A)** Average withdrawal threshold response to mechanical stimulation after injection with EGFP-AAV (black) or shKLHL1-AAV (red) at low titer of shKLHL1 or EGFP-AAV. **(B)** The average response from delivery of high titer shKLHL1-AAV or EGFP-AAV virus, *p ≤ 0.006.

Baseline behavioral tests were performed a week prior to injection in all mice (untreated). Blinded experimental measurements started 4–5 days after AAV injection and were performed 2–3 times weekly for 3 weeks thereafter (Figure 7). As seen in all figures, baseline withdrawal threshold values were indistinguishable in mice injected with either EGFP-AAV or shKLHL1-AAV at both titers, trial 1: EGFP-AAV, 5.2 g vs. 5.0 g for shKLHL1-AAV2; *n* = 7, *p* = 0.2; trial 2: EGFP-AAV, 5.2 g vs. 5.5 g in shKLHL1-AAV; *n* = 11, *p* = 0.2).

AAV injections caused some pain and inflammation, as expected (Ishihara et al., 2012), resulting in lower threshold values after injection compared with untreated values, as seen after injection in trial 1 at weeks 1–3 compared to untreated. Overall, the doses of shKLHL1 delivered in trial 1 exerted no effect on mechanical sensitivity, as seen in Figure 7A; the individual weekly averages were: week 1: EGFP-AAV, 4.4 ± 0.4 g vs. 4.1 ± 0.3 g for shKLHL1-AAV-2 (*n* = 7, *p* = 0.2); week 2: EGFP-AAV, 4.0 ± 0.2 g vs. 4.2 ± 0.2 g for shKLHL1-AAV-2 (*n* = 7, *p* = 0.3); and week 3: EGFP-AAV = 4.2 ± 0.2 g vs. 4.7 ± 0.1 g in shKLHL1-AAV-2 (*n* = 7, *p* = 0.056).

The dose delivered in trial 2 (~9.0 × 10^10^ viral particles, Figure 7B) induced significant differences in mechanical threshold values in shKLHL1-injected mice after 1 week. Unlike trial 1 and trial 2 control conditions, mechanical thresholds in shKLHL1-injected mice did not decrease compared to untreated conditions and they were significantly higher than their corresponding controls at all times tested. Week 1: EGFP-AAV, 3.6 ± 0.3 g vs. 4.6 ± 0.5 g for shKLHL1-AAV-1 (*n* = 11, *p* = 0.006); week 2: EGFP-AAV, 3.8 ± 0.5 g vs. 5.8 ± 0.3 g for shKLHL1-AAV-1 (*n* = 11, *p* = 0.006); and week 3: EGFP-AAV, 3.5 ± 0.4 g vs. 5.6 ± 0.5 g for shKLHL1-AAV-1 (*n* = 11, *p* = 0.001).

**Figure 8:**
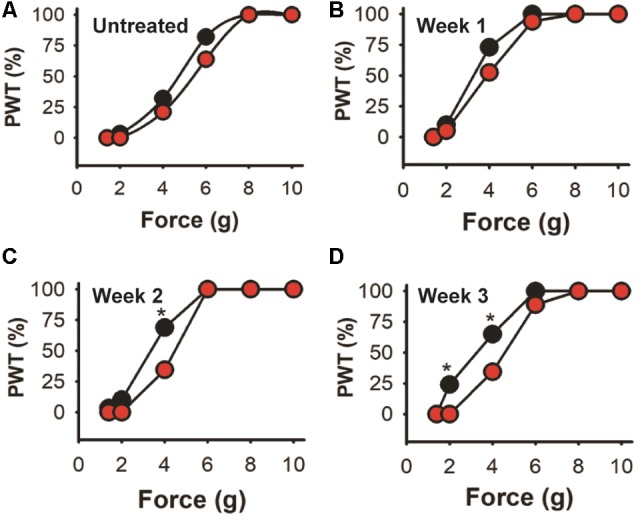
Cumulative response in the high dose trial. Data from trial 2 expressed as the cumulative response in the mice population after a given stimulus. **(A)** Untreated mice. **(B–D)** Responses after 1, 2 and 3 weeks of EGFP-AAV (black symbols) and shKLHL1-AAV (red symbols) injection, *p < 0.05.

Further analysis is shown in Figure 8 where trial 2 data is shown as the percentage of the mice population displaying paw withdrawal threshold (PWT %) at a given Von Frey filament force value. There are no significant differences in baseline values (A) or after the first week after injection (B) between the experimental and control-treated mice populations (Kolmogorov–Smirnov test). However, 69% of EGFP-AAV injected mice responded to the 4 g von Frey filament stimulus 2 weeks after injection (C) in comparison with only 34% in the shKLHL1-AAV injected population (*p* < 0.05). This difference was more pronounced after 3 weeks of injection (D), with changes between the two populations at 4 g (35% shKLHL1-AAV vs. 64% EGFP-AAV) and 2 g (0% shKLHL1-AAV vs. 24% EGFP-AAV). Note that all mice were more sensitive after injections as a result of the AAV injections (compare to untreated).

## Discussion

T-type Ca_V_3.2 channel up-regulation is associated with diabetic neuropathy (Jagodic et al., 2007), neuropathic pain (Choi et al., 2016) and irritable bowel syndrome (Marger et al., 2011). Similarly, increased Ca_V_3.2 function is found in chemotherapy-induced toxic neuropathies, and their inhibition with T-type calcium channels blockers decreases pain sensitivity (Flatters and Bennett, 2004; Okubo et al., 2011; Li et al., 2017). Also, paracetamol fails to induce analgesic effects in Ca_V_3.2 knockout mice, suggesting these channels are necessary for analgesic actions (Kerckhove et al., 2014).

KLHL1 protein is a constitutive modulator of Ca_V_3.2 channels, and here we show that in its absence, KLHL1 KO mice elicit increased mechanical sensitivity threshold (decreased sensitivity to pain). Thus, KLHL1 could have significant potential as a molecular target to modulate neuropathic pain, akin to the effect of the auxiliary subunit α_2_δ’s role on HVA channels (Field et al., 2006; Nguyen et al., 2009). KLHL1 functions in an analogous manner as the α_2_δ subunit, which is targeted by GBP and similar drugs resulting in a significant decrease of Ca_V_2.2 currents in part by a reduction of functional channels at the plasma membrane (Vega-Hernández and Felix, 2002; Field et al., 2006; Hendrich et al., 2008; Aromolaran et al., 2009, 2010, 2012; Martínez-Hernández et al., 2011).

KLHL1 KO neurons displayed decreased T-type calcium current density due to the down-regulation of Ca_V_3.2, they also displayed decreased DRG neuron excitability, in line with the absence of KLHL1. Partial blockade of T-type channels with 100 nM NCC 55–0396 in WT neurons reduced their excitability to a comparable level to that of the KO DRGs, suggesting T-type channel down-regulation is solely responsible for the decrease in excitability.

Similar to other studies (Shin et al., 2003; Wang et al., 2015), we found Ca_V_3.1 expression is detectable in DRG neurons from mice (in contrast to studies performed in rats, which report Ca_V_3.1 is absent in DRG neurons from that species (Talley et al., 1999; Wen et al., 2006), however, it is well established that Ca_V_3.1 does not have a functional role in DRGs, and accordingly, RNAseq data, Allen Atlas data and our own ICC data (not shown) demonstrate Ca_V_3.1 expression in DRG is much lower in mice (if present) compared to Ca_V_3.2. The fact that Ca_V_3.1 is not physiologically relevant in DRG neurons possibly explains our observation that this channel type was not upregulated in KLHL1 KO DRG neurons, in contrast with our observations in hippocampal neurons from KLHL1 KO (Perissinotti et al., 2014). Similarly, KLHL1 also interacts with Ca_V_2.1, and their levels were also unaffected in this system (in contrast to KLHL1 KO hippocampal neurons). The cause of this differential regulation is not known yet but may again be because Ca_V_2.1 is not physiologically relevant in DRG neurons, thus suggesting tissue-specific modulatory mechanisms are tuned to control the expression of functionally relevant channels and their isoforms differently in central nervous system (CNS) vs. DRG- neurons (Zamponi and Snutch, 2013).

Administration of T-type calcium channels blockers such as mibefradil or ethosuximide *via* intraperitoneal or paw injection, or chronic intrathecal infusion show reversal of neuropathic pain in rats (Dogrul et al., 2003; Chen et al., 2015); similarly, knockdown of Ca_V_3.2 resulted in decreased analgesic effect (Bourinet et al., 2005, 2016). Here, we were able to alter mechanical sensitivity in WT mice by knocking down KLHL1 levels in DRG neurons using anti-KLHL1 shRNA AAV injected into the hind paws of WT mice. We were able to sample the decreased expression of KLHL1 and Ca_V_3.2 T-type channels by Western Blot analysis in a sample of three pooled L4 DRGs injected with shKLHL1 AAV compared to an EGFP AAV- injected DRGs sample. von Frey filament tests confirmed that mice whose hind paws were injected with shKLHL1 were less sensitive to pain than those injected with control EGFP-AAV (5.6 ± 0.5 g threshold compared to 3.5 ± 0.4 g, respectively at week 3 of treatment). These values are in line with the majority of data in the literature (Watanabe et al., 2015; Garcia-Caballero et al., 2016; M’Dahoma et al., 2016; Stemkowski et al., 2016; Ogawa et al., 2018); however, two groups have reported withdrawal threshold values around 1 g (Costigan et al., 2009; Chiu et al., 2013; Vicuna et al., 2015; Choi et al., 2016). It is no clear the reason for these differences, given that most studies were done using C57B/6 mice (6–14 weeks old).

*In vitro* data from neuronal cultures indicated that viral titers of less than 5.0 × 10^10^ particles would be less efficient, here we found we found that *in vivo* delivery of 5.1 × 10^10^ shKLHL1-AAV vector genomes was the minimal titer that exerted an effect, although it was only attained after 3 weeks post-treatment. In contrast, delivery of 9.0 × 10^10^ vector genomes of virus-containing shKLHL1 sequences was efficacious at increasing the mice’s withdrawal threshold, demonstrating reduced sensitivity to pain. Alternative delivery routes such as subcutaneous or intramuscular injections are also known as viable options, and can be assessed in the future (Towne et al., 2009).

In summary, our study shows that KLHL1 is a physiological modulator Ca_V_3.2 expression and function in DRG neurons and that KLHL1 may be a viable molecular target to reduce pain transmission by lowering Ca_V_3.2 expression. Modulation of neuronal excitability by alteration of KLHL1 levels and/or function may represent a novel method of treatment for neuropathic disorders and may help facilitate the development of novel therapeutic alternatives.

## Data Availability Statement

The raw data supporting the conclusions of this article will be made available by the authors, without undue reservation, to any qualified researcher.

## Ethics Statement

All animal studies presented in this study were reviewed and approved by an independent Institutional Animal Care and Use Committee (IACUC) at Loyola University Chicago Stritch School of Medicine.

## Author Contributions

EM-H performed electrophysiology, biochemistry and behavioral pain experiments and wrote the article. AZ and EA performed behavioral pain experiments. PP performed electrophysiology experiments. YH and MK generated the KLHL1 KO mouse. JM designed and produced all viral constructs and AAVs. EP-R performed biochemistry and ICC experiments, designed experiments, directed the research, and revised the manuscript.

## Conflict of Interest

EP-R and JM are authors of the US Patent 10,047,377. The remaining authors declare that the research was conducted in the absence of any commercial or financial relationships that could be construed as a potential conflict of interest.

## References

[B1] Andrade A. Denome S. Jiang Y. Q. Marangoudakis S. Lipscombe D. (2010). Opioid inhibition of N-type Ca^2+^ channels and spinal analgesia couple to alternative splicing. Nat. Neurosci. 13, 1249–1256. 10.1038/nn.2643 20852623 PMC2956429

[B2] Aromolaran K. A. Benzow K. A. Cribbs L. L. Koob M. D. Piedras-Renteria E. S. (2009). Kelch-like 1 protein upregulates T-type currents by an actin-F dependent increase in α(1H) channels *via* the recycling endosome. Channels 3, 402–412. 10.4161/chan.3.6.9858 19806008

[B3] Aromolaran K. A. Benzow K. A. Cribbs L. L. Koob M. D. Piedras-Renteria E. S. (2010). T-type current modulation by the actin-binding protein Kelch-like 1. Am. J. Physiol. Cell Physiol. 298, C1353–C1362. 10.1152/ajpcell.00235.2009 20147652

[B4] Aromolaran K. A. Benzow K. A. Cribbs L. L. Koob M. D. Piedras-Renteria E. S. (2012). Elimination of the actin-binding domain in kelch-like 1 protein induces T-type calcium channel modulation only in the presence of action potential waveforms. J. Signal. Transduct. 2012:505346. 10.1155/2012/505346 22848812 PMC3401526

[B5] Basbaum A. I. Bautista D. M. Scherrer G. Julius D. (2009). Cellular and molecular mechanisms of pain. Cell 139, 267–284. 10.1016/j.cell.2009.09.028 19837031 PMC2852643

[B6] Bellampalli S. S. Ji Y. Moutal A. Cai S. Wijeratne E. M. K. Gandini M. A.. (2019). Betulinic acid, derived from the desert lavender Hyptis emoryi, attenuates paclitaxel-, HIV-, and nerve injury-associated peripheral sensory neuropathy *via* block of N- and T-type calcium channels. Pain 160, 117–135. 10.1097/j.pain.0000000000001385 30169422 PMC6309937

[B7] Bernal Sierra Y. A. Haseleu J. Kozlenkov A. Bégay V. Lewin G. R. (2017). Genetic tracing of Ca_v_3.2 T-type calcium channel expression in the peripheral nervous system. Front. Mol. Neurosci. 10:70. 10.3389/fnmol.2017.00070 28360836 PMC5350092

[B8] Bonin R. P. Bories C. De Koninck Y. (2014). A simplified up-down method (SUDO) for measuring mechanical nociception in rodents using von Frey filaments. Mol. Pain 10:26. 10.1186/1744-8069-10-26 24739328 PMC4020614

[B9] Bourinet E. Alloui A. Monteil A. Barrère C. Couette B. Poirot O.. (2005). Silencing of the Ca_v_3.2 T-type calcium channel gene in sensory neurons demonstrates its major role in nociception. EMBO J. 24, 315–324. 10.1038/sj.emboj.7600515 15616581 PMC545807

[B10] Bourinet E. Francois A. Laffray S. (2016). T-type calcium channels in neuropathic pain. Pain 157, S15–S22. 10.1097/j.pain.0000000000000469 26785151

[B11] Cain S. M. Snutch T. P. (2010). Contributions of T-type calcium channel isoforms to neuronal firing. Channels 4, 475–482. 10.4161/chan.4.6.14106 21139420 PMC3052247

[B12] Chaplan S. R. Bach F. W. Pogrel J. W. Chung J. M. Yaksh T. L. (1994). Quantitative assessment of tactile allodynia in the rat paw. J. Neurosci. Methods 53, 55–63. 10.1016/0165-0270(94)90144-9 7990513

[B13] Chen Y. L. Tsaur M. L. Wang S. W. Wang T. Y. Hung Y. C. Lin C. S.. (2015). Chronic intrathecal infusion of mibefradil, ethosuximide and nickel attenuates nerve ligation-induced pain in rats. Br. J. Anaesth. 115, 105–111. 10.1093/bja/aev198 26089446

[B14] Chiu I. M. Heesters B. A. Ghasemlou N. Von Hehn C. A. Zhao F. Tran J.. (2013). Bacteria activate sensory neurons that modulate pain and inflammation. Nature 501, 52–57. 10.1038/nature12479 23965627 PMC3773968

[B15] Choi S. Na H. S. Kim J. Lee J. Lee S. Kim D.. (2007). Attenuated pain responses in mice lacking Ca_V_3.2 T-type channels. Genes Brain Behav. 6, 425–431. 10.1111/j.1601-183x.2006.00268.x 16939637

[B16] Choi S. Yu E. Hwang E. Llinás R. R. (2016). Pathophysiological implication of Ca_V_3.1 T-type Ca^2+^ channels in trigeminal neuropathic pain. Proc. Natl. Acad. Sci. U S A 113, 2270–2275. 10.1073/pnas.1600418113 26858455 PMC4776481

[B17] Coste B. Crest M. Delmas P. (2007). Pharmacological dissection and distribution of NaN/Nav1.9, T-type Ca^2+^ currents, and mechanically activated cation currents in different populations of DRG neurons. J. Gen. Physiol. 129, 57–77. 10.1085/jgp.200609665 17190903 PMC2151607

[B18] Costigan M. Moss A. Latremoliere A. Johnston C. Verma-Gandhu M. Herbert T. A.. (2009). T-cell infiltration and signaling in the adult dorsal spinal cord is a major contributor to neuropathic pain-like hypersensitivity. J. Neurosci. 29, 14415–14422. 10.1523/JNEUROSCI.4569-09.2009 19923276 PMC2813708

[B19] Cribbs L. L. Lee J. H. Yang J. Satin J. Zhang Y. Daud A.. (1998). Cloning and characterization of α1H from human heart, a member of the T-type Ca^2+^ channel gene family. Circ. Res. 83, 103–109. 10.1161/01.res.83.1.103 9670923

[B20] Davies A. Hendrich J. Van Minh A. T. Wratten J. Douglas L. Dolphin A. C. (2007). Functional biology of the α_2_δ subunits of voltage-gated calcium channels. Trends Pharmacol. Sci. 28, 220–228. 10.1016/j.tips.2007.03.005 17403543

[B21] Dogrul A. Gardell L. R. Ossipov M. H. Tulunay F. C. Lai J. Porreca F. (2003). Reversal of experimental neuropathic pain by T-type calcium channel blockers. Pain 105, 159–168. 10.1016/s0304-3959(03)00177-5 14499432

[B22] Dubreuil A. S. Boukhaddaoui H. Desmadryl G. Martinez-Salgado C. Moshourab R. Lewin G. R.. (2004). Role of T-type calcium current in identified D-hair mechanoreceptor neurons studied *in vitro*. J. Neurosci. 24, 8480–8484. 10.1523/JNEUROSCI.1598-04.2004 15456821 PMC6729907

[B23] Field M. J. Cox P. J. Stott E. Melrose H. Offord J. Su T. Z.. (2006). Identification of the α2-δ-1 subunit of voltage-dependent calcium channels as a molecular target for pain mediating the analgesic actions of pregabalin. Proc. Natl. Acad. Sci. U S A 103, 17537–17542. 10.1073/pnas.0409066103 17088553 PMC1859964

[B24] Flatters S. J. Bennett G. J. (2004). Ethosuximide reverses paclitaxel- and vincristine-induced painful peripheral neuropathy. Pain 109, 150–161. 10.1016/j.pain.2004.01.029 15082137

[B25] Florio V. Striessnig J. Catterall W. A. (1992). Purification and reconstitution of skeletal muscle calcium channels. Meth. Enzymol. 207, 529–546. 10.1016/0076-6879(92)07037-o 1382201

[B26] Gandini M. A. Sandoval A. Felix R. (2014). Whole-cell patch-clamp recordings of Ca^2+^ currents from isolated neonatal mouse dorsal root ganglion (DRG) neurons. Cold Spring Harb. Protoc. 2014, 389–395. 10.1101/pdb.prot073205 24692487

[B27] Garcia-Caballero A. Gadotti V. M. Chen L. Zamponi G. W. (2016). A cell-permeant peptide corresponding to the cUBP domain of USP5 reverses inflammatory and neuropathic pain. Mol. Pain 12:1744806916642444. 10.1177/1744806916642444 27130589 PMC4955966

[B28] Garcia-Caballero A. Gadotti V. M. Stemkowski P. Weiss N. Souza I. A. Hodgkinson V.. (2014). The deubiquitinating enzyme USP5 modulates neuropathic and inflammatory pain by enhancing Ca_v_3.2 channel activity. Neuron 83, 1144–1158. 10.1016/j.neuron.2014.07.036 25189210

[B29] Gee N. S. Brown J. P. Dissanayake V. U. Offord J. Thurlow R. Woodruff G. N. (1996). The novel anticonvulsant drug, gabapentin (Neurontin), binds to the α2δ subunit of a calcium channel. J. Biol. Chem. 271, 5768–5776. 10.1074/jbc.271.10.5768 8621444

[B30] Hendrich J. Van Minh A. T. Heblich F. Nieto-Rostro M. Watschinger K. Striessnig J.. (2008). Pharmacological disruption of calcium channel trafficking by the α2δ ligand gabapentin. Proc. Natl. Acad. Sci. U S A 105, 3628–3633. 10.1073/pnas.0708930105 18299583 PMC2265195

[B31] Ishihara A. Bartlett J. S. Bertone A. L. (2012). Inflammation and immune response of intra-articular serotype 2 adeno-associated virus or adenovirus vectors in a large animal model. Arthritis 2012:735472. 10.1155/2012/735472 22288012 PMC3263587

[B32] Jagodic M. M. Pathirathna S. Joksovic P. M. Lee W. Nelson M. T. Naik A. K.. (2008). Upregulation of the T-type calcium current in small rat sensory neurons after chronic constrictive injury of the sciatic nerve. J. Neurophysiol. 99, 3151–3156. 10.1152/jn.01031.2007 18417624 PMC2667888

[B33] Jagodic M. M. Pathirathna S. Nelson M. T. Mancuso S. Joksovic P. M. Rosenberg E. R.. (2007). Cell-specific alterations of T-type calcium current in painful diabetic neuropathy enhance excitability of sensory neurons. J. Neurosci. 27, 3305–3316. 10.1523/JNEUROSCI.4866-06.2007 17376991 PMC6672477

[B34] Kerckhove N. Mallet C. François A. Boudes M. Chemin J. Voets T.. (2014). Ca_v_3.2 calcium channels: the key protagonist in the supraspinal effect of paracetamol. Pain 155, 764–772. 10.1016/j.pain.2014.01.015 24447516

[B35] Kohlbrenner E. Henckaerts E. Rapti K. Gordon R. E. Linden R. M. Hajjar R. J.. (2012). Quantification of AAV particle titers by infrared fluorescence scanning of coomassie-stained sodium dodecyl sulfate-polyacrylamide gels. Hum. Gene Ther. Methods 23, 198–203. 10.1089/hgtb.2012.049 22816378 PMC4015068

[B36] Li Y. Tatsui C. E. Rhines L. D. North R. Y. Harrison D. S. Cassidy R. M.. (2017). Dorsal root ganglion neurons become hyperexcitable and increase expression of voltage-gated T-type calcium channels (Ca_v3_.2) in paclitaxel-induced peripheral neuropathy. Pain 158, 417–429. 10.1097/j.pain.0000000000000774 27902567 PMC5303135

[B37] Liu Q. Y. Chen W. Cui S. Liao F. F. Yi M. Liu F. Y.. (2019). Upregulation of Ca_v_3.2 T-type calcium channels in adjacent intact L4 dorsal root ganglion neurons in neuropathic pain rats with L5 spinal nerve ligation. Neurosci. Res. 142, 30–37. 10.1016/j.neures.2018.04.002 29684385

[B38] Marger F. Gelot A. Alloui A. Matricon J. Ferrer J. F. Barrere C.. (2011). T-type calcium channels contribute to colonic hypersensitivity in a rat model of irritable bowel syndrome. Proc. Natl. Acad. Sci. U S A 108, 11268–11273. 10.1073/pnas.1100869108 21690417 PMC3131334

[B39] Martínez-Hernández E. Sandoval A. González-Ramírez R. Zoidis G. Felix R. (2011). Inhibition of recombinant N-type and native high voltage-gated neuronal Ca^2+^ channels by AdGABA: mechanism of action studies. Toxicol. Appl. Pharmacol. 250, 270–277. 10.1016/j.taap.2010.10.030 21059371

[B40] Martins D. F. Prado M. R. Daruge-Neto E. Batisti A. P. Emer A. A. Mazzardo-Martins L.. (2015). Caffeine prevents antihyperalgesic effect of gabapentin in an animal model of CRPS-I: evidence for the involvement of spinal adenosine A1 receptor. J. Peripher. Nerv. Syst. 20, 403–409. 10.1111/jns.12149 26456872

[B41] M’Dahoma S. Gadotti V. M. Zhang F. X. Park B. Nam J. H. Onnis V.. (2016). Effect of the T-type channel blocker KYS-05090S in mouse models of acute and neuropathic pain. Pflugers Arch. 468, 193–199. 10.1007/s00424-015-1733-1 26354962

[B42] Melrose H. L. Kinloch R. A. Cox P. J. Field M. J. Collins D. Williams D. (2007). [_3_H] pregabalin binding is increased in ipsilateral dorsal horn following chronic constriction injury. Neurosci. Lett. 417, 187–192. 10.1016/j.neulet.2007.02.068 17367933

[B43] Nguyen D. Deng P. Matthews E. A. Kim D. S. Feng G. Dickenson A. H.. (2009). Enhanced pre-synaptic glutamate release in deep-dorsal horn contributes to calcium channel α-2-δ-1 protein-mediated spinal sensitization and behavioral hypersensitivity. Mol. Pain 5:6. 10.1186/1744-8069-5-6 19216737 PMC2646710

[B44] Ogawa N. Terashima T. Oka K. Chan L. Kojima H. (2018). Gene therapy for neuropathic pain using dorsal root ganglion-targeted helper-dependent adenoviral vectors with GAD67 expression. Pain Rep. 3:e695. 10.1097/pr9.0000000000000695 30706038 PMC6344132

[B45] Okubo K. Takahashi T. Sekiguchi F. Kanaoka D. Matsunami M. Ohkubo T.. (2011). Inhibition of T-type calcium channels and hydrogen sulfide-forming enzyme reverses paclitaxel-evoked neuropathic hyperalgesia in rats. Neuroscience 188, 148–156. 10.1016/j.neuroscience.2011.05.004 21596106

[B46] Perez-Reyes E. Cribbs L. L. Daud A. Lacerda A. E. Barclay J. Williamson M. P.. (1998). Molecular characterization of a neuronal low-voltage-activated T-type calcium channel. Nature 391, 896–900. 10.1016/s0166-2236(98)01331-9 9495342

[B47] Perissinotti P. P. Ethington E. A. Almazan E. Martínez-Hernández E. Kalil J. Koob M. D.. (2014). Calcium current homeostasis and synaptic deficits in hippocampal neurons from Kelch-like 1 knockout mice. Front. Cell. Neurosci. 8:444. 10.3389/fncel.2014.00444 25610372 PMC4285801

[B48] Pradhan A. D. Case A. M. Farrer R. G. Tsai S. Y. Cheatwood J. L. Martin J. L.. (2010). Dendritic spine alterations in neocortical pyramidal neurons following postnatal neuronal Nogo-A knockdown. Dev. Neurosci. 32, 313–320. 10.1159/000309135 20938157 PMC3021499

[B49] Sarkey J. P. Chu M. McShane M. Bovo E. Ait Mou Y. Zima A. V.. (2011). Nogo-A knockdown inhibits hypoxia/reoxygenation-induced activation of mitochondrial-dependent apoptosis in cardiomyocytes. J. Mol. Cell. Cardiol. 50, 1044–1055. 10.1016/j.yjmcc.2011.03.004 21420413 PMC3091973

[B50] Shin J. B. Martinez-Salgado C. Heppenstall P. A. Lewin G. R. (2003). A T-type calcium channel required for normal function of a mammalian mechanoreceptor. Nat. Neurosci. 6, 724–730. 10.1038/nn1076 12808460

[B52] Stemkowski P. García-Caballero A. Gadotti V. M. M’Dahoma S. Huang S. Black S. A. G.. (2016). TRPV1 nociceptor activity initiates USP5/T-type channel-mediated plasticity. Cell Rep. 17, 2901–2912. 10.1016/j.celrep.2016.11.047 27974205

[B53] Suárez L. M. Suárez F. Del Olmo N. Ruiz M. González-Escalada J. R. Solís J. M. (2005). Presynaptic NMDA autoreceptors facilitate axon excitability: a new molecular target for the anticonvulsant gabapentin. Eur. J. Neurosci. 21, 197–209. 10.1111/j.1460-9568.2004.03832.x 15654857

[B54] Takahashi T. Aoki Y. Okubo K. Maeda Y. Sekiguchi F. Mitani K.. (2010). Upregulation of Ca(v)3.2 T-type calcium channels targeted by endogenous hydrogen sulfide contributes to maintenance of neuropathic pain. Pain 150, 183–191. 10.1016/j.pain.2010.04.022 20546998

[B55] Talley E. M. Cribbs L. L. Lee J. H. Daud A. Perez-Reyes E. Bayliss D. A. (1999). Differential distribution of three members of a gene family encoding low voltage-activated (T-type) calcium channels. J. Neurosci. 19, 1895–1911. 10.1523/JNEUROSCI.19-06-01895.1999 10066243 PMC6782581

[B56] Todorovic S. M. Jevtovic-Todorovic V. (2011). T-type voltage-gated calcium channels as targets for the development of novel pain therapies. Br. J. Pharmacol. 163, 484–495. 10.1111/j.1476-5381.2011.01256.x 21306582 PMC3101611

[B57] Towne C. Pertin M. Beggah A. T. Aebischer P. Decosterd I. (2009). Recombinant adeno-associated virus serotype 6 (rAAV2/6)-mediated gene transfer to nociceptive neurons through different routes of delivery. Mol. Pain 5:52. 10.1186/1744-8069-5-52 19737386 PMC2747840

[B58] Vega-Hernández A. Felix R. (2002). Down-regulation of N-type voltage-activated Ca^2+^ channels by gabapentin. Cell. Mol. Neurobiol. 22, 185–190. 10.1023/a:1019865822069 12363200 PMC11533754

[B59] Vicuna L. Strochlic D. E. Latremoliere A. Bali K. K. Simonetti M. Husainie D.. (2015). The serine protease inhibitor SerpinA3N attenuates neuropathic pain by inhibiting T cell-derived leukocyte elastase. Nat. Med. 21, 518–523. 10.1038/nm.3852 25915831 PMC4450999

[B60] Wang X. L. Tian B. Huang Y. Peng X. Y. Chen L. H. Li J. C.. (2015). Hydrogen sulfide-induced itch requires activation of Ca_v_3.2 T-type calcium channel in mice. Sci. Rep. 5:16768. 10.1038/srep16768 26602811 PMC4658482

[B61] Watanabe M. Ueda T. Shibata Y. Kumamoto N. Shimada S. Ugawa S. (2015). Expression and regulation of Cav3.2 T-type calcium channels during inflammatory hyperalgesia in mouse dorsal root ganglion neurons. PLoS One 10:e0127572. 10.1371/journal.pone.0127572 25974104 PMC4431781

[B62] Weiss N. Zamponi G. W. (2017). Trafficking of neuronal calcium channels. Neuronal Signal. 1:NS20160003 10.1042/ns20160003 PMC7373241 32714572

[B63] Weiss N. Zamponi G. W. (2019). T-type channel druggability at a crossroads. ACS Chem. Neurosci. 10, 1124–1126. 10.1021/acschemneuro.9b00031 30697997

[B64] Wen X. J. Li Z. J. Chen Z. X. Fang Z. Y. Yang C. X. Li H.. (2006). Intrathecal administration of Ca_v_3.2 and Ca_v_3.3 antisense oligonucleotide reverses tactile allodynia and thermal hyperalgesia in rats following chronic compression of dorsal root of ganglion. Acta Pharmacol. Sin. 27, 1547–1552. 10.1111/j.1745-7254.2006.00461.x 17112407

[B65] White G. Lovinger D. M. Weight F. F. (1989). Transient low-threshold Ca^2+^ current triggers burst firing through an afterdepolarizing potential in an adult mammalian neuron. Proc. Natl. Acad. Sci. U S A 86, 6802–6806. 10.1073/pnas.86.17.6802 2549548 PMC297934

[B66] Zamponi G. W. Snutch T. P. (2013). Advances in voltage-gated calcium channel structure, function and physiology. Biochim. Biophys. Acta 1828:1521. 10.1016/j.bbamem.2013.03.014 23518035

[B67] Zolotukhin S. Byrne B. J. Mason E. Zolotukhin I. Potter M. Chesnut K.. (1999). Recombinant adeno-associated virus purification using novel methods improves infectious titer and yield. Gene Ther. 6, 973–985. 10.1038/sj.gt.3300938 10455399

